# Machine learning identifies a compact gene set for monitoring the circadian clock in human blood

**DOI:** 10.1186/s13073-017-0406-4

**Published:** 2017-02-28

**Authors:** Jacob J. Hughey

**Affiliations:** Department of Biomedical Informatics, Vanderbilt University School of Medicine, Nashville, TN USA

**Keywords:** Circadian, Machine learning, Precision medicine, Meta-analysis, Transcriptome

## Abstract

**Background:**

The circadian clock and the daily rhythms it produces are crucial for human health, but are often disrupted by the modern environment. At the same time, circadian rhythms may influence the efficacy and toxicity of therapeutics and the metabolic response to food intake. Developing treatments for circadian dysfunction, as well as optimizing the daily timing of treatments for other health conditions, will require a simple and accurate method to monitor the molecular state of the circadian clock.

**Methods:**

Here we used a recently developed method called ZeitZeiger to predict circadian time (CT, time of day according to the circadian clock) from genome-wide gene expression in human blood.

**Results:**

In cross-validation on 498 samples from 60 individuals across three publicly available datasets, ZeitZeiger predicted CT in single samples with a median absolute error of 2.1 h. The predictor trained on all 498 samples used 15 genes, only two of which are part of the core circadian clock. By then applying ZeitZeiger to 475 additional samples from the same three datasets, we quantified how the circadian clock in the blood was affected by various perturbations to the sleep–wake and light–dark cycles. Finally, we extended ZeitZeiger (1) to handle intra-individual variation by making predictions based on multiple samples taken a known time apart, and (2) to handle inter-individual variation by personalizing predictions based on samples from the respective individual. Each of these strategies improved prediction of CT by ~20%.

**Conclusions:**

Our results are an important step towards precision circadian medicine. In addition, our generalizable extensions to ZeitZeiger may be applicable to the growing number of biological datasets that contain multiple observations per individual.

**Electronic supplementary material:**

The online version of this article (doi:10.1186/s13073-017-0406-4) contains supplementary material, which is available to authorized users.

## Background

Much of human physiology, from sleep to immune function, has a daily rhythm [1]. Driving many of these rhythms is a system of molecular oscillators, called circadian clocks, that is active in nearly every tissue in the body [2] and that senses and entrains to daily rhythms in our environment [3, 4]. In animal models, disrupting the circadian system can have a wide range of phenotypic consequences [5–7]. In humans, circadian dysfunction is linked to a number of health conditions, including cancer [8], major depressive disorder [9], and obesity [10]. At least some of the circadian dysfunction in humans seems to be a result of multiple features of the modern environment, e.g. shift work and reduced exposure to sunlight [11, 12]. Consequently, improving circadian function by photic, behavioral, or other means, which has been called chronomedicine, could greatly benefit human health [13].

At the same time, increasing evidence suggests that circadian rhythms influence the efficacy and toxicity of therapeutics [14, 15] as well as the metabolic consequences of food intake [16]. For example, over half of the 100 best-selling drugs in the U.S. target a protein whose messenger RNA (mRNA) in mice shows a circadian rhythm in at least one organ [17]. Using knowledge of the body’s circadian rhythms to optimize the timing of interventions has been called chronotherapy [18, 19].

Large-scale implementation of chronotherapy may be more difficult than first thought, however, as evidence suggests that at any given time of day, different individuals’ circadian rhythms are at different points in the cycle. For instance, the circadian phase of entrainment (as measured by the Munich Chronotype Questionnaire) varies highly between individuals [20], as well as with age and between day-workers and shift-workers [21]. Furthermore, the circadian phase of clock gene expression in hair follicle cells is correlated with morningness/eveningness preference [22]. These observations imply that the optimal timing of a given intervention may vary from one person to another.

Thus, one critical component of both chronotherapy and chronomedicine, which together might be called precision circadian medicine, is a method to monitor the state of a person’s circadian clock(s). For chronotherapy, such a method would be an input; for chronomedicine, an output. Unfortunately, current methods for monitoring the clock in humans have limitations. One such method is to measure the sleep–wake rhythm, either by questionnaires, sleep logs, or actigraphy [23]. Although measuring sleep–wake is non-invasive and has led to valuable insights [24, 25], sleep is also influenced by non-circadian processes and has a complex relationship with the various clocks throughout the body [26].

Another method to assess the state of the clock is to measure melatonin in plasma or saliva. In particular, dim light melatonin onset (DLMO) is the gold standard for circadian phase [27–29]. Determining DLMO, however, requires collecting many samples under controlled conditions over at least several hours, making it impractical for widespread use or for monitoring the circadian clock in real time. Furthermore, DLMO only reflects the phase of the central clock in the suprachiasmatic nucleus (which controls secretion of melatonin by the pineal gland), making it unable to report on clocks in other tissues. Efforts to address these limitations have shown promise, but studies so far have included only a small number of individuals and have measured either a small set of pre-selected genes (which may not be optimal) [30] or a large number of metabolites by mass spectrometry (which limits the potential for wide application) [31].

One resource for robust and efficient biomarker discovery is publicly available “omics” data [32, 33]. Although there are now multiple publicly available datasets of the circadian transcriptome in human blood, these data have not yet been integrated to develop a marker of the circadian clock.

We recently developed a supervised learning method called ZeitZeiger, which can learn to predict a periodic variable (e.g. time of day) from a high-dimensional observation [34]. In our initial study, we used ZeitZeiger to train a predictor of circadian time (CT) from transcriptome data in mice. The predictor, which was based on the expression of only 13 genes, achieved state-of-the-art accuracy and also detected when the circadian clock was phase-shifted or dysfunctional. Given ZeitZeiger’s success at determining the state of the clock in mice, we wondered how it would perform on data from humans.

Here we applied ZeitZeiger to three publicly available datasets of circadian transcriptome data from human blood. We found that ZeitZeiger learned to use a small set of genes to accurately predict the CT of a single sample. This allowed ZeitZeiger to detect how circadian gene expression is affected by various perturbations to the light–dark and sleep–wake cycles. We then investigated two ways to improve prediction accuracy: first, by using groups of samples, and second, by combining the initial prediction with that from a personal predictor trained only on samples from the respective individual. Our results are an important step towards precision circadian medicine.

## Methods

### Processing time of day and other metadata

The nomenclature for time of day in chronobiology is complicated [35]. Complicating our analysis even further, each of the three datasets used a different experimental design (Table 1) and not all of the datasets included individual-level information for DLMO (which would indicate the phase of the central clock).

**Table 1:** Datasets of circadian gene expression in human blood

Dataset	Ref.	Control condition	Perturbation condition	Participants	Samples (control; perturbation)	Interval	Age (mean ± sd)	Female
GSE39445	[42]	Constant dim (after LD 16:8)	7 days of sleep restriction	24	221; 217	3 h	27.5 ± 4.3 y	42%
GSE48113	[36]	Dim:dark 14.7:9.3 (after LD 16:8)	4 28-h days (forced desynchrony)	22	147; 139	4 h	26.3 ± 3.4 y	50%
GSE56931	[43]	LD 14:10	1 night of sleep deprivation	14	130; 119	4 h	29.7 ± 8.9 y	57%

“Dim” corresponds to <10 lux in GSE39445 and <5 lux in GSE48113

For both GSE48113 and GSE56931, the first samples were collected after participants had been in the lab no more than one day. For GSE39445, the first samples were collected after participants had been in the lab for nine days, but for each participant, the midpoint of sleep opportunities in the lab coincided with the midpoint of sleep in that participant’s habitual sleep–wake schedule. In all three datasets, then, the phase of each participant’s circadian clock should be based primarily on the natural light–dark cycle. Therefore, we calculated the time of day for each sample in each dataset (e.g. 08:00) relative to sunrise time, using either the dates and geographic location provided by the authors (GSE56931) or the average sunrise time in the respective geographic location (GSE39445 and GSE48113). We refer to this adjusted time of day as “circadian time.”

For GSE48113, because DLMO for each participant in each condition was not provided, we calculated “time relative to DLMO” using the average DLMO for each condition (21:59 for “in phase,” 23:03 for “out of phase”), as provided in the original publication [36].

Unless otherwise specified, we used only the samples from the control condition in each dataset. This corresponded to “sleep extension” in GSE39445, “in phase with respect to melatonin” in GSE48113, and “baseline” in GSE56931.

### Processing gene expression data

Gene expression from the three microarray datasets was processed using MetaPredict [37] (https://github.com/jakejh/metapredict), which maps probes to Entrez Gene IDs (where necessary, summarizing the expression of multiple probes using the median), performs intra-study normalization and log-transformation, and uses ComBat [38] to perform cross-study normalization. The merged data of control samples from all three datasets consisted of 17,477 genes measured in 498 samples.

### Using ZeitZeiger to predict CT

ZeitZeiger is a supervised learning method for periodic variables, i.e. variables that are continuous and bounded and for which the maximum value is equivalent to the minimum value (e.g. the angle in polar coordinates between 0 and 2π). ZeitZeiger uses the training observations to learn a sparse representation of the variation associated with the periodic variable, then makes a prediction for a test observation using maximum likelihood [34].

Training a ZeitZeiger predictor involves the following steps: (1) fitting a periodic smoothing spline to the intensity of each feature (e.g. the expression of each gene) as a function of the periodic variable [39]; (2) discretizing and scaling the spline fits; (3) using the discretized and scaled fits to calculate sparse principal components (SPCs; linear combinations of a small set of features) [40]; and (4) fitting a periodic smoothing spline to the intensity of each SPC as a function of the periodic variable. Making predictions involves two steps: (1) projecting the test observation from feature-space to SPC-space; and (2) using the spline fits of the SPCs from the training data to perform maximum likelihood estimation. The two main parameters of ZeitZeiger are sumabsv and nSPC. The former corresponds to the amount of *L*_1_ regularization used to calculate the SPCs, while the latter corresponds to the number of SPCs used for prediction. Other parameters of ZeitZeiger include the number of knots for spline fitting and the number of time-points for discretization. In this study, we always used three knots (which constrains the spline’s flexibility and makes it more resistant to noise) and 12 time-points.

Tenfold cross-validation was performed such that all samples from a given individual were in the same fold. The folds were identical when predicting CT for groups of samples and when training universal predictors to provide universal guidance to the personal predictors in leave-one-sample-out cross-validation. Because only three datasets were available (two of which were from the same research group), we elected not to perform leave-one-study-out cross-validation or to have a separate group of validation samples from one or more of the datasets. Instead, we only performed tenfold cross-validation across all controls samples from all three datasets. This means we may be underestimating generalization error (perhaps cancelling out the imperfect standardization of time of day), but also makes it simpler to use all the control samples when testing strategies for improving accuracy.

We did use a leave-one-study-out strategy when analyzing the effects of sleep–wake perturbations. For each dataset in turn, we trained a ZeitZeiger predictor (sumabsv = 2 and nSPC = 2) on only the control samples from two datasets, then tested on all samples (control and “treatment”) from the third. Thus, prediction accuracies from this analysis are not directly comparable to those from tenfold cross-validation, but within this analysis, one can still compare results for control and treatment samples within each dataset.

The signal-to-noise ratio of circadian rhythmicity for gene *j* was calculated as

$$ S N{R}_j=\frac{max{f}_j(t)- min{f}_j(t)}{s_j}, $$

where *f*_*j*_(*t*) is the expression of gene *j* as a function of time *t* and *s*_*j*_ is the root mean squared error of the periodic spline fit.

### Providing universal guidance when training personal predictors

**Fig. 1:**
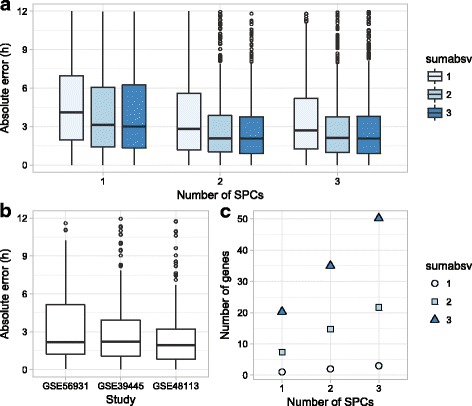
Using ZeitZeiger to predict circadian time in control samples from three datasets (tenfold cross-validation). **a** *Boxplots* of absolute error for various values of sumabsv (regularization parameter) and nSPC (number of SPCs). **b** *Boxplots* of absolute error for each dataset at sumabsv = 2 and nSPC = 2. **c** Mean number of genes in the predictors from cross-validation for various values of sumabsv and nSPC

For each fold of tenfold cross-validation (performed across individuals) and each sample of personal leave-one-sample-out cross-validation (performed on samples from a single individual), our procedure for universal guidance worked as follows. First, samples from the other nine folds (the universal training set) were used to train a “universal” predictor (the same predictor used for tenfold cross-validation in Fig. 1). Next, the training samples from the current individual (i.e. the personal training set) were filtered to include only those genes used in the universal predictor, resulting in the shrunken personal training set. Thus, universal guidance here exploits the fact that ZeitZeiger performs feature selection. Finally, the shrunken personal training set was used to train the personal predictor.

### Extending ZeitZeiger for multiple samples and multiple predictors

When making a prediction for a single sample *x*, ZeitZeiger calculates the log-likelihood *L*(*t*|*x*), where *t* ∈ [0, 1) is the scaled periodic variable (e.g. time of day). The predicted time $\widehat{t}$ is then

$$ \widehat{t}=\underset{t\in \left[0,1\right)}{ \arg \max } L\left( t\Big| x\right). $$

Now suppose we have a group of *n* samples, and for each sample, we have the measurements *x*_*i*_ and a time difference *τ*_*i*_, which is the time of the *i* th sample relative to the time of a particular sample in the group. In the simplest case, *n* = 1 and *τ* = 0. For two samples taken anti-phase to each other, we could have *τ*_1_ = 0 and *τ*_2_ = 0.5. Now we can combine the log-likelihood for each sample and make one prediction for the entire group as follows:

$$ \widehat{t}=\underset{t\in \left[0,1\right)}{ \arg \max }{\displaystyle \sum_{i=1}^n L\left(\left( t+{\tau}_i\right) \mod 1\Big|{x}_i\right)}, $$

where the *mod* operator means that times less than 0 or greater than 1 “wrap around” to be between 0 and 1, and $\widehat{t}$ is the estimated time at which *τ* = 0.

Combining predictors can be done in two ways, the first of which works similarly to combining samples. Suppose we have a group of *m* predictors, where for a given sample *x*, *L*_*j*_(*t*|*x*) is the log-likelihood for predictor *j*. For the situation of one universal and one personal predictor, *m* = 2. The ensemble prediction is then

$$ \widehat{t}=\underset{t\in \left[0,1\right)}{ \arg \max }{\displaystyle \sum_{j=1}^m{L}_j\left( t\Big| x\right).} $$

The second way to combine predictors is to use the circular mean. In this case, the ensemble prediction is

$$ \widehat{t}=\frac{1}{2\pi} atan2\left({\displaystyle \sum_{j=1}^m sin\left(2\pi {\widehat{t}}_j\right)},{\displaystyle \sum_{j=1}^m cos\left(2\pi {\widehat{t}}_j\right)}\right). $$

This second way is simpler and, on our data, provides a slightly larger improvement in accuracy. Therefore, all ensemble predictions in this study are based on the circular mean.

Our current implementations implicitly weight each sample or each predictor equally, but one could imagine incorporating explicit weights into any of these calculations, then learning the weights through an additional round of cross-validation.

### Calculating phase differences in clock gene expression

Phase differences in clock gene expression between control and perturbation conditions (Additional file 1: Figure S4) were calculated as previously described [41]. Briefly, if *f*(*t*) is the spline fit of expression versus time for a given gene in a given condition, we estimated phase as the time of peak expression, i.e. *argmax f*(*t*). Calculation of phase differences accounted for the fact that *t* is periodic, e.g. CT2 is 4 h ahead of CT22.

## Results

### Predicting CT of single samples in three datasets

We assembled three publicly available datasets of genome-wide gene expression in human blood (Table 1) [36, 42, 43]. Each dataset consisted of samples taken throughout the day from individuals in a control condition and a condition in which sleep and the light–dark cycle were perturbed. In the original publications, two of the three perturbations were found to shift the phase of melatonin secretion (for the third, melatonin was not measured) [36, 42]. Therefore, to establish a baseline for the clock in blood cells, we focused first on the control samples (although even the control conditions in each study were not identical). We merged and batch-corrected the gene expression measurements [37, 38] and standardized the time of day values (see “Methods”).

Using the combined data, we then performed tenfold cross-validation, in which ZeitZeiger learned to predict a sample’s CT based on its gene expression. We ran cross-validation with a range of values for ZeitZeiger’s two main parameters, sumabsv (which controls the amount of regularization) and nSPC (which controls how many SPCs are used for prediction). Samples from the same individual were always in the same fold.

We evaluated the results of cross-validation in terms of absolute error (absolute difference between predicted and observed CT; Fig. 1a). The median absolute error achieved by the optimal parameter values was 2.1 h (interquartile range, 2.8 h). The expected absolute error of a random predictor is 6 h. Similar to our experience predicting CT using gene expression in mice [34], prediction accuracy plateaued at sumabsv = 2 and nSPC = 2. Prediction accuracy was similar across the three datasets (Fig. 1b). On average, the predictors from cross-validation trained with sumabsv = 2 and nSPC = 2 were based on the expression of 15 genes (Fig. 1c). These results suggest that ZeitZeiger can use the expression of a small number of genes to accurately predict CT from a single sample of human blood.

**Fig. 2:**
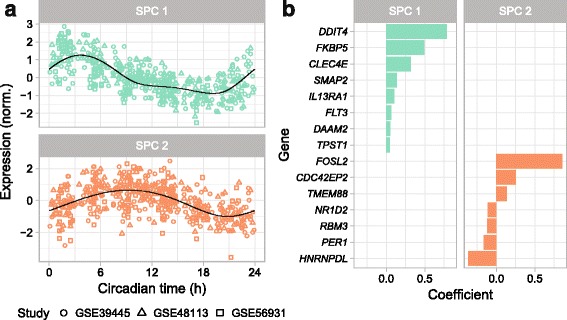
Properties of the predictor trained on all control samples from the three datasets (sumabsv = 2, nSPC = 2). **a** Expression of the two SPCs vs. circadian time. Each point is a sample. *Black curves* correspond to periodic smoothing splines fit by ZeitZeiger. The signal-to-noise ratios of the two SPCs are 3.06 and 2.01, respectively (compare to Additional file 1: Figure S1). **b** Genes and coefficients for the two SPCs. Genes are sorted by their respective coefficients. The expression of a given SPC in a given sample corresponds to the dot product of the coefficients for that SPC and the gene expression for that sample

To examine the circadian patterns that ZeitZeiger was learning, we used the parameter values sumabsv = 2 and nSPC = 2 to train a predictor on all control samples from the three datasets. The SPCs calculated by ZeitZeiger, each of which is a linear combination of genes, are designed to explain variation in gene expression associated with CT. The predictor’s two SPCs showed times of peak expression that were shifted from each other by ~6 h (Fig. 2a), similar to the multi-organ predictor of CT that we trained on gene expression in mice [34]. Interestingly, however, the expression of SPC 1 as a function of CT was markedly non-sinusoidal. Moreover, of the 15 genes that formed the two SPCs (Fig. 2b), only two, *NR1D2* (*REV-ERBβ*) and *PER1*, are thought to be part of the core circadian clock. Consistent with this observation, the signal-to-noise ratio of circadian rhythmicity was generally lower for clock genes than for the 15 genes in the predictor (Additional file 1: Figure S1). When we allowed ZeitZeiger to predict CT using only core clock genes, absolute error on cross-validation increased by a median of 27% (*P* = 7 × 10^–6^ by paired Wilcoxon rank-sum test; Additional file 1: Figure S2), demonstrating ZeitZeiger’s ability to select the most informative genes.

### Analyzing the effects of perturbations to sleep and light–dark cycles

Having established a baseline for predicting CT in human blood, we next investigated how predictions of CT were affected by the perturbation condition in each dataset. Here we followed a leave-one-study-out strategy, in which we trained a predictor (sumabsv = 2 and nSPC = 2) on control samples from two datasets, then applied the predictor to control and perturbation samples from the third dataset.

**Fig. 3:**
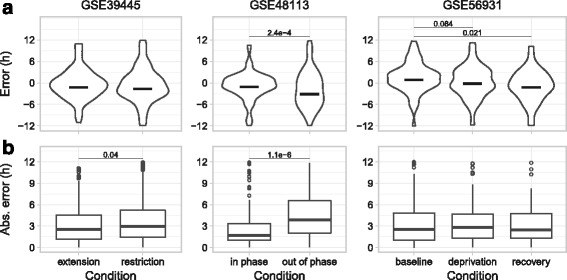
Applying ZeitZeiger to gene expression from perturbations of the sleep–wake and light–dark cycles. For each of the three datasets, a predictor was trained on control samples from the other two datasets, then tested on all samples from the dataset of interest. **a** *Violin plots* of error and (**b**) *boxplots* of absolute error for each condition in each dataset. Numbers above the plots indicate *P* values < 0.1 (circular ANOVA for error and Wilcoxon rank-sum test for absolute error). For ease of visualization, *boxplots* of absolute error do not show three outliers in GSE39445, one in GSE48113, and three in GSE56931. The left-most condition in each dataset is the control. In GSE39445, “restriction” refers to seven nights of 6-h sleep opportunity. In GSE48113, “out of phase” refers to the forced-desynchrony protocol in which sleep is out of phase with melatonin and the central clock. In GSE56931, “deprivation” refers to one full day of sleep deprivation and “recovery” refers to a normal sleep opportunity the next night

The perturbations had several effects on predictions of CT (Fig. 3 and Additional file 1: Figure S3). First, six days of restricted sleep opportunity (GSE39445) worsened prediction accuracy by 16%, consistent with weaker circadian oscillations in gene expression [42]. Second, the forced-desynchrony protocol (GSE48113), which causes the central clock to go into free-run [36, 44], induced an apparent phase delay of 2 h relative to the original light–dark cycle and increased variability in prediction error by 42% (based on circular standard deviation). Third, a single night of sleep deprivation with the lights on (GSE56931) induced an apparent phase delay of 2.1 h, consistent with previous findings on the effect of sleep deprivation and light on circadian phase in humans [45–47]. These effects on predictions of CT, which were based primarily on the expression of non-core clock genes (Fig. 2), were largely consistent with the expression of core clock genes (Additional file 1: Figure S4). In addition, the phase delays in predicted CT induced by sleep restriction (GSE39445) and forced-desynchrony protocol (GSE48113) were similar, but not identical, to the corresponding delays in DLMO (Additional file 1: Table S1), which suggests that the circadian clock in blood cells may respond to these perturbations slightly differently than the central circadian clock in the brain.

To verify our results for the forced-desynchrony protocol (GSE48113), we performed cross-validation using the control and perturbation (“in phase” and “out of phase”) samples together, in this case predicting time relative to average DLMO in each condition (see “Methods”). This analysis gave similar results as before (Additional file 1: Figure S5A–C). In addition, variability in prediction error for “out of phase” samples remained high, even when performing cross-validation using only the “out of phase” samples (Additional file 1: Figure S6), consistent with previous observations that the forced-desynchrony protocol disrupts circadian gene expression in the blood [36]. The predictor trained on all samples from GSE48113 (sumabsv = 2 and nSPC = 2) included many of the same genes that were in the predictor trained on control samples from all three datasets (Additional file 1: Figure S5D, E). We therefore focused on the control samples for the remainder of our analysis.

### Predicting CT using multiple samples

Although ZeitZeiger was originally designed to predict the CT of a single sample, we wondered if prediction accuracy could be improved by using multiple samples from the same individual. We therefore extended ZeitZeiger to make predictions for groups of samples, where the time difference between each sample in the group is known (see “Methods”). For each individual in the three datasets, we then constructed groups of two samples (taken either 8–9 h or 12 h apart) or three samples (taken over 12 h). We predicted the CT of each group in tenfold cross-validation using sumabsv = 2 and nSPC = 2 (group information is only used during testing, not during training).

**Fig. 4:**
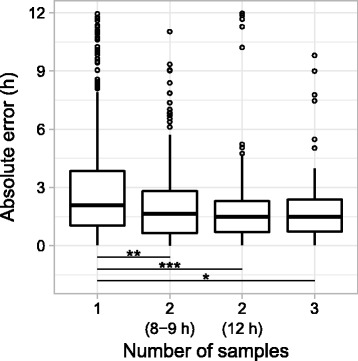
Predicting CT using various numbers of control samples from the same individual. *Boxplots* of absolute error from tenfold cross-validation. From left to right, the numbers of groups (one prediction per group) were 498, 198, 200, and 121. Levels of statistical significance (Wilcoxon rank-sum test): * *P* = 2.5 × 10^–4^, ** *P* = 1.9 × 10^–5^, *** *P* = 5.9 × 10^–7^. Other differences were not statistically significant

Compared to predictions based on a single sample, predictions based on two samples taken either 8–9 h apart were ~21% more accurate (0.43 h reduction in median absolute error; Fig. 4). Predictions based on two samples taken 12 h apart or on three samples showed a slight additional increase in accuracy (*P* > 0.3 by Wilcoxon rank-sum test). These results suggest that ZeitZeiger can use multiple relatively noisy samples to make better predictions.

### Personalizing predictions of CT

Because we previously found that ZeitZeiger can learn to make accurate predictions even given small training sets with low time resolution [34], we wondered if ZeitZeiger could learn to accurately predict CT given only the samples from a single individual (~8 control samples per individual in the three datasets). To test this, we performed leave-one-sample-out cross-validation for each individual (sumabsv = 2 and nSPC = 2). Unfortunately, the predictions from personal cross-validation were only slightly better than random and much worse than those from the original tenfold cross-validation (Additional file 1: Figure S7).

**Fig. 5:**
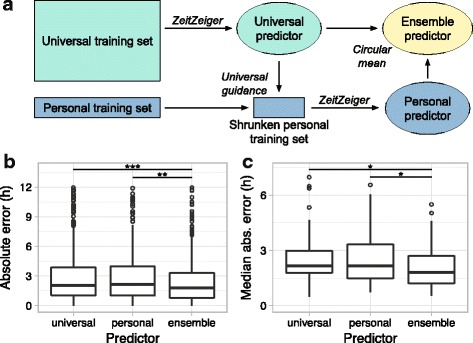
Personalized prediction of CT in control samples from the three datasets. All predictors were trained using sumabsv = 2 and nSPC = 2. Levels of statistical significance (paired Wilcoxon rank-sum test): * *P* < 10^–3^, ** *P* = 7.4 × 10^–5^, *** *P* = 5.9 × 10^–11^. **a** *Schematic* of procedure for training personal and ensemble predictors with universal guidance. For the training sets, the height represents observations and the width represents features (e.g. genes). Universal guidance refers to filtering for only those genes used by the universal predictor. **b** *Boxplots* of absolute error for universal (standard tenfold cross-validation), personal (leave-one-sample-out cross-validation for each individual), and ensemble (circular mean of universal and personal) predictors. **c** *Boxplots* of median absolute error (by individual) for universal, personal, and ensemble predictors

Given that the merged dataset used throughout this paper included the expression of 17,477 genes, we suspected that ~8 samples per individual might not be enough to prevent ZeitZeiger from overfitting. We therefore devised a procedure for training personal predictors with “universal guidance,” which removes all features (i.e. genes) from the personal training set except for those selected by the “universal” predictor (i.e. the predictor trained on samples from multiple other individuals; see “Methods” and Fig. 5a).

Using universal guidance, the personal predictors achieved similar accuracy on leave-one-sample-out cross-validation to the universal predictors on tenfold cross-validation (Fig. 5b, c). We then combined the universal and personal predictors into an ensemble using the circular mean (see “Methods” and Fig. 5a). Strikingly, the ensemble predictor was ~20% more accurate than either single predictor, both on a per-sample and per-individual basis (Fig. 5b, c and Additional file 1: Figure S8). The improvement in accuracy was robust for predictions based on at least seven personal training samples (Additional file 1: Figure S9). Applying this strategy of ensemble learning to groups of samples did not improve accuracy further, likely due to the small number of samples in the personal training sets (Additional file 1: Figure S10). Taken together, these results suggest that predictions based on a large training set from multiple individuals can be fine-tuned by predictions based on a carefully constructed training set from the individual of interest.

To further investigate the differences in circadian gene expression between individuals, we employed our strategy for universal guidance to train one predictor for each individual. We found that the subset of genes selected by ZeitZeiger (from the 15 genes in the universal predictor, as shown in Fig. 2) varied from one personal predictor to another (Additional file 1: Figure S11A). Furthermore, although the difference between peak times of SPC 1 and SPC 2 was largely consistent across individuals (Additional file 1: Figure S11B), the actual value of peak times was not (Additional file 1: Figure S11C). These results suggest that even the 15 “consensus” genes show meaningful interindividual variation in circadian expression.

## Discussion

Developing treatments that improve the function of or that account for the circadian system has the potential to improve multiple areas of human health. Realizing this potential, however, requires a robust method for monitoring an individual’s circadian rhythm. Here we developed a predictor of CT in human blood by applying machine learning to genome-wide gene expression. We demonstrated accurate prediction for single samples using a small set of genes, then developed two strategies that each improved accuracy by ~20%.

Both strategies rely on having multiple observations per individual. The first strategy, combining samples taken a known time apart, uses them at the prediction step in order to deal with measurement noise and intra-individual variation. The second strategy, combining universal and personal predictors, uses them at the training step in order to deal with inter-individual variation. An important component of the second strategy was universal guidance, which uses feature selection in the universal predictor to limit the variance of the personal predictor. Conceptually, our strategy for personalizing predictions is similar to an approach called customized training, which involves finding training observations that look similar to a given test observation [48]. Given current technology, the requirement for at least six samples per individual is impractical. In the future, however, it may be possible to personalize predictions using samples from multiple individuals (e.g. those with similar phases of entrainment) and by combining tissue-based measurements with actigraphy.

Comparing our current results in human blood to our previous results in multiple mouse organs, two main differences emerge. First, our predictions here are less accurate, a consequence of circadian gene expression in humans being noisier (although here we analyzed only blood, we have observed similar levels of noise in human brain [41]). This increased noise is likely due to genetic, environmental, and tissue-specific factors (circadian gene expression in mouse blood has not been measured). In addition, some datasets from mice are based on tissue pooled from multiple animals, so a fair interspecies comparison of the variation in circadian gene expression in a given tissue has yet to be made.

Second, in contrast to the predictor we developed in mice, most of the genes in the human blood-based predictor are not thought to be part of the core circadian clock. This difference is likely due to the fact that the mouse predictor was trained on data from 12 organs, which discouraged ZeitZeiger from selecting genes whose circadian expression was tissue-specific (the vast majority of genes [17]) and resulted in a strong enrichment for core clock genes. In this study, the dominant gene for SPC 1 was DDIT4 (REDD1), which encodes a protein that inhibits mTOR signaling as part of the response to cellular stress [49]. The dominant gene for SPC 2 was FOSL2 (FRA2), which encodes a subunit of the AP-1 transcription factor and is therefore involved in numerous aspects of cell proliferation [50].

Because only 2/15 genes in the predictor are part of the core clock and because circadian rhythms can be “masked” by direct effects of the environment, it seems reasonable to wonder if the predictor learned by ZeitZeiger is truly a reflection of the circadian clock. For multiple reasons, we believe it is. First, the control condition in the sleep restriction dataset (GSE39445) was a constant routine that minimized diurnal variation in sleep and feeding [42]. Second, we obtained a similar set of genes when analyzing the control samples from the three datasets compared to analyzing control and perturbation samples from the forced-desynchrony dataset (GSE48113). Third, 4/10 core clock genes had a signal-to-noise in the top 0.5% of all genes, suggesting that the circadian system is a major driver of the observed rhythmicity in the blood transcriptome. Fourth, both the top genes in the predictor, DDIT4 and FOSL2, show circadian rhythms in expression in rodents (in animals entrained to light–dark, then released into constant darkness) [51, 52]. Furthermore, circadian expression of FOSL2 in rat pineal gland is dependent on the central clock in the suprachiasmatic nucleus [51]. Although more work is needed to elucidate the mechanistic details, these results suggest that expression of the 13 non-core clock genes in the predictor is regulated by the circadian clock.

If ZeitZeiger is capturing the progression of the clock, as we believe it is, then our findings suggest that the forced-desynchrony protocol, which decouples sleep–wake from the body’s central clock [36], may also cause a misalignment of ~1 h between the central clock and the clock in blood cells. This misalignment, especially if it affects clocks in peripheral tissues besides the blood, may be relevant to the adverse metabolic and cardiovascular effects caused by the forced-desynchrony protocol [53, 54].

One limitation of this study is that, because not all of the datasets included individual-level information about DLMO, we had no direct measurement for the internal time of each individual’s central circadian clock. Consequently, we trained ZeitZeiger to predict the externally measured time of day. Some of the inaccuracy of the predictions could therefore be due to interindividual variation in the alignment of external and internal time of day, i.e., the phase of entrainment. Such variation could explain why the personal predictors in the ensemble improved accuracy: they helped adjust for each individual’s circadian phase. Before our approach can be used clinically, it will need to be validated in prospective studies. Such studies will likely involve testing ZeitZeiger and/or the 15-gene set alongside melatonin and actigraphy outside the laboratory setting.

## Conclusions

Although here we have focused on genome-wide gene expression in blood, our methodology can be applied to any type of data from any tissue. We are therefore hopeful that in addition to its utility in chronotherapy and chronomedicine, ZeitZeiger will support efforts to study how the circadian system integrates information from multiple environmental cues [13], how circadian function is altered in pathophysiological conditions, and to develop biomarkers for sleep-related and circadian-related disorders [55]. Furthermore, as the number of observations for each individual increases, e.g. in electronic medical records, our framework for personalizing predictions may prove useful in many areas of precision medicine.

## Additional file

Additional file 1: Supplementary figures and table. (PDF 1212 kb)

## Abbreviations

CT: Circadian time
DLMO: Dim light melatonin onset
SPC: Sparse principal component
