# Supplementary material for: Machine learning identifies a compact gene set for monitoring the circadian clock in human blood
Source: Genome Med. 2017 Feb 28;9:19. doi: 10.1186/s13073-017-0406-4 (PMC5329904; doi:10.1186/s13073-017-0406-4)
Supplement: Additional file 1: — Supplementary figures and table. (PDF 1212 kb) [file 13073_2017_406_MOESM1_ESM.pdf]

Supplementary figures and table for:

# Machine learning identifies a compact gene set for monitoring the circadian clock in human blood

Jacob J. Hughey*

Department of Biomedical Informatics, Vanderbilt University School of Medicine, Nashville, TN, USA

Figure S1

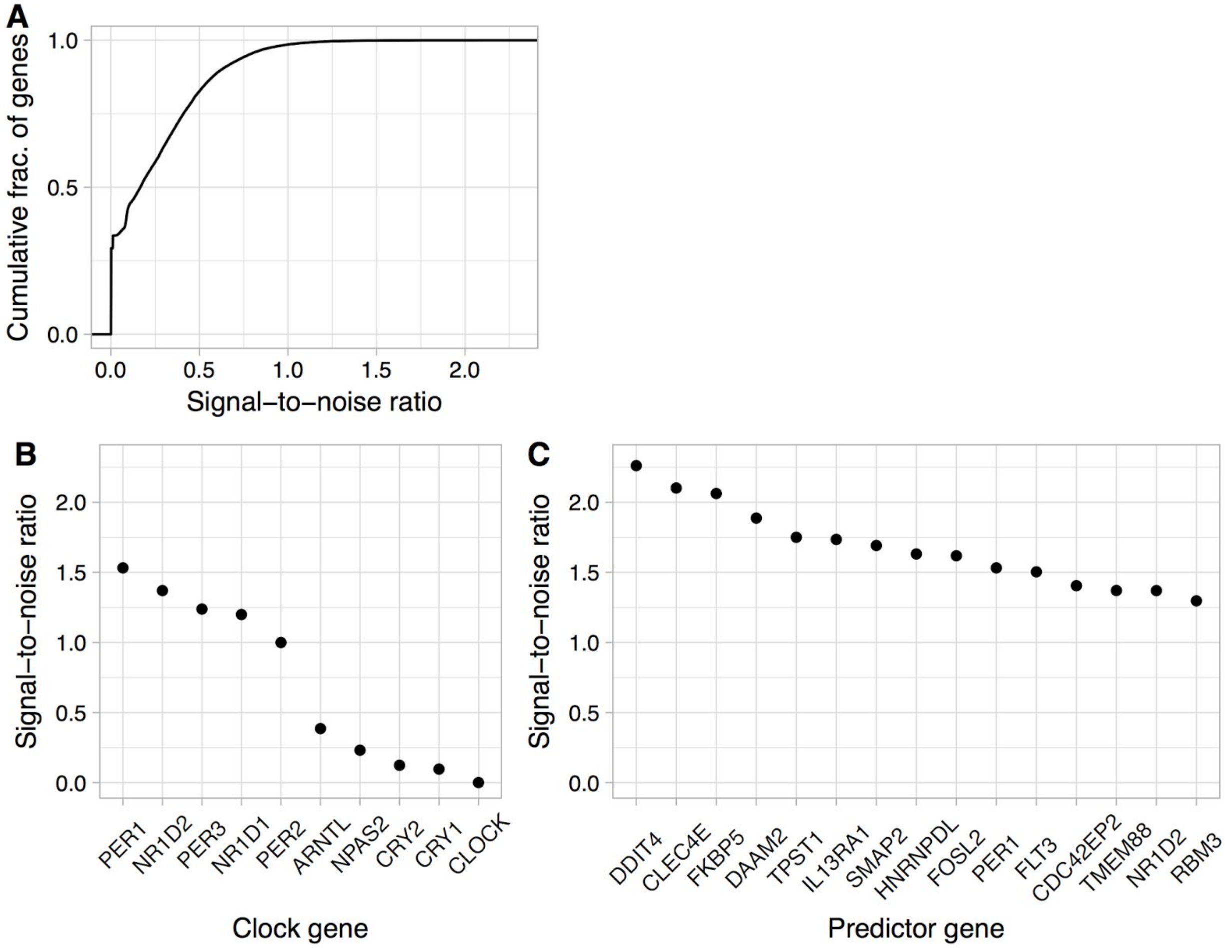


Strength of circadian rhythmicity, quantified as a signal-to-noise ratio (SNR), for gene expression in human blood. As in Fig. 2, data is from control samples from all three datasets. **(A)** Cumulative distribution function of SNR for all genes. **(B)** SNR for core clock genes. **(C)** SNR for genes in the ZeitZeiger predictor.

Figure S2

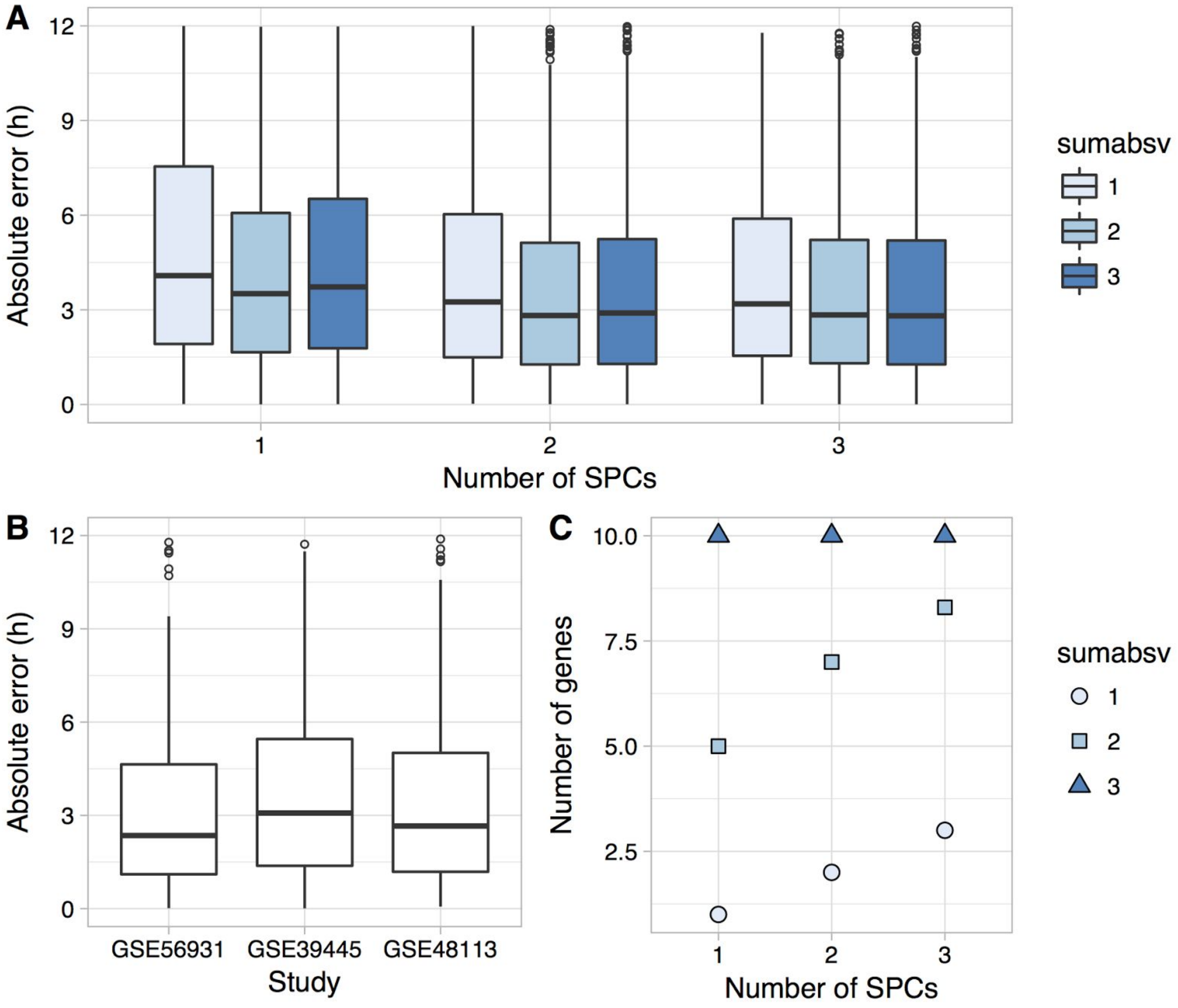


Ten-fold cross-validation to predict CT using only the core clock genes (otherwise identical to Fig. 1).

Figure S3

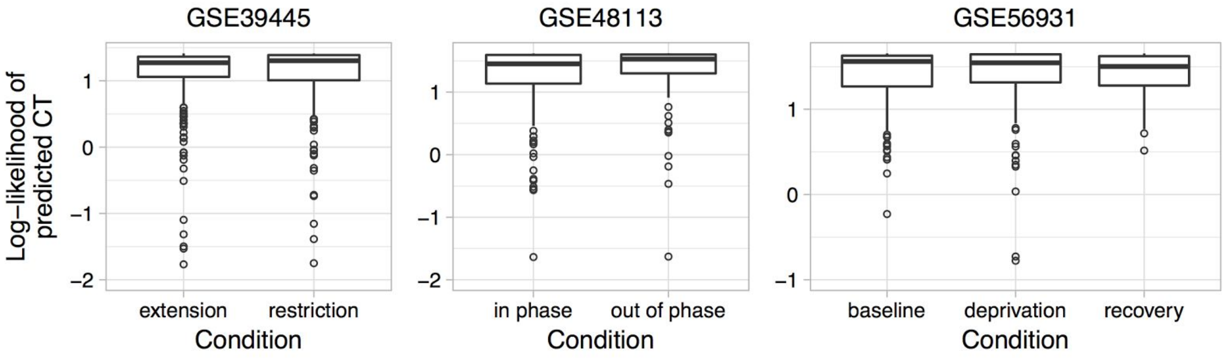


Boxplots of log-likelihood of predicted circadian time for each condition in each dataset (related to Fig. 3). For each of the three datasets, a predictor was trained on control samples from the other two datasets, then tested on all samples from the dataset of interest. The left-most condition in each dataset is the control.

## Figure S4

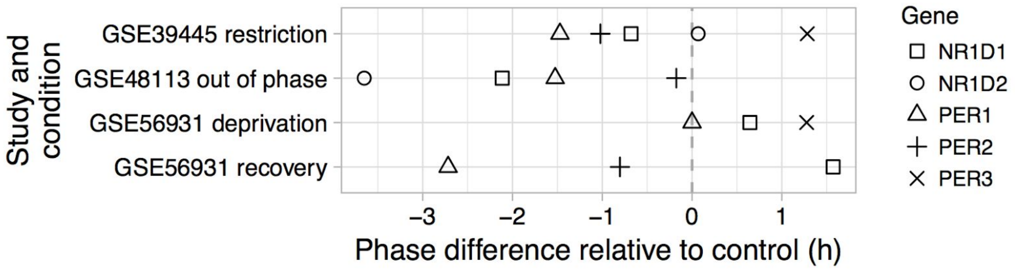


Phase difference (i.e., difference in circadian time of peak expression) between each perturbation condition and the respective control condition for core clock genes. A negative phase difference corresponds to a delay relative to the control 24-h light-dark cycle. Points are only shown if the clock gene showed a signal-to-noise ratio of at least 0.4 in both the control condition and the perturbation condition.

## Table S1

| Dataset | Mean delay in DLMO (h) | Mean delay in predicted CT (h) |
|---|---|---|
| GSE39445 | 0.77 | 0.36 |
| GSE48113 | 1.07 | 1.99 |

Phase shifts between control and treatment conditions (relative to the original 24-h cycle) in two datasets. Mean shifts in DLMO were obtained from the original publications. Note that DLMO is based on multiple time-points per subject per condition.

## Figure S5

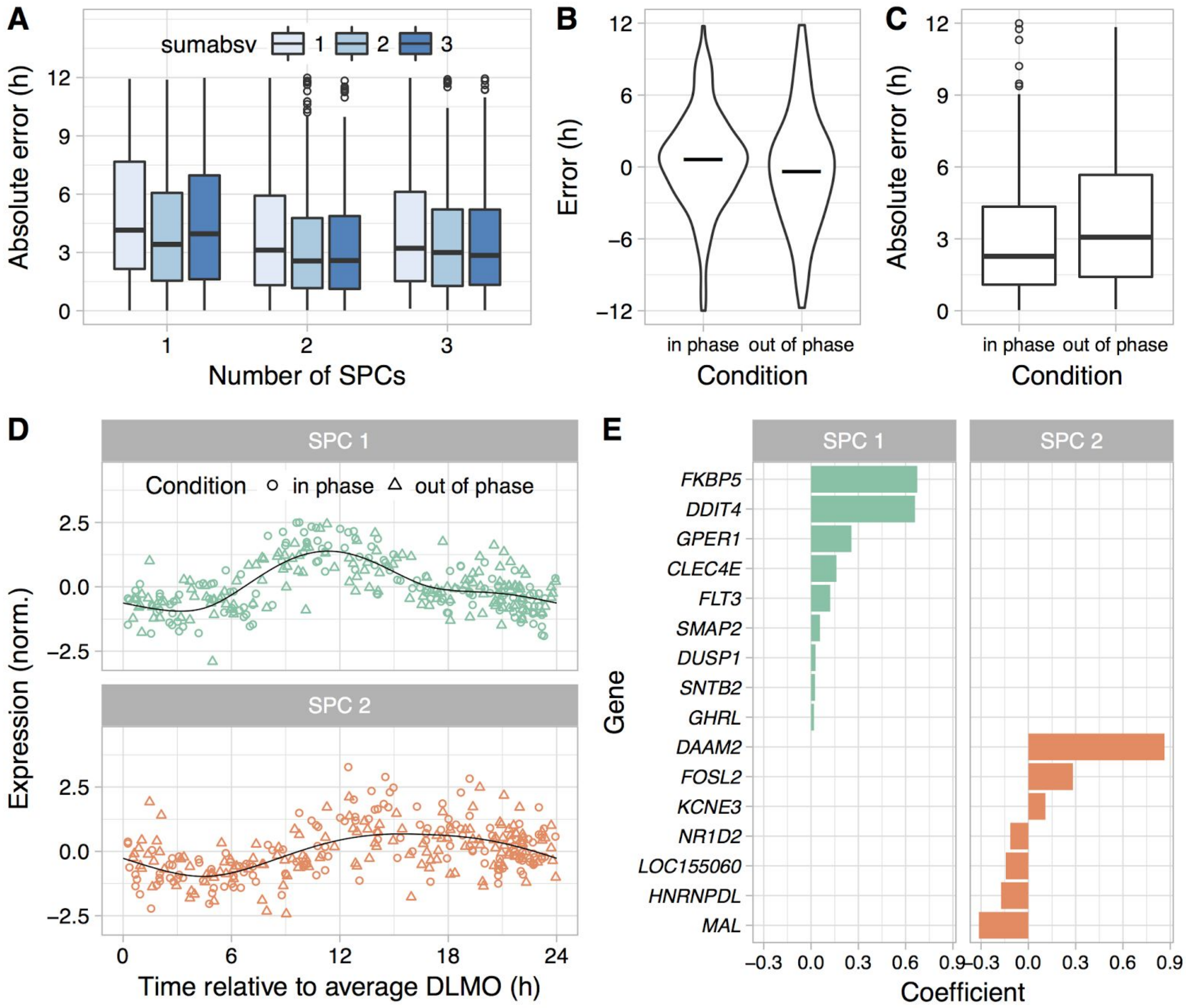


Applying ZeitZeiger to all samples (“in phase” and “out of phase”) from GSE48113. Instead of predicting circadian time, ZeitZeiger was trained to predict time relative to average DLMO in each condition. Average DLMO was ~1 h later (relative to the original light-dark cycle) in the “out of phase” samples than in the “in phase” samples, so using time relative to DLMO instead of CT merely shifts the times in the “out of phase” samples and reduces the apparent delay between “in phase” and “out of phase” samples by ~1 h. **(A)** Boxplots of absolute error on 10-fold cross-validation for various parameter values. **(B)** Error and **(C)** absolute error on cross-validation (sumabsv=2 and nSPC=2) for each condition. **(D)** Expression of the two SPCs vs. time relative to DLMO (sumabsv=2). Each point is a sample. Black curves correspond to periodic smoothing splines fit by ZeitZeiger. **(E)** Genes and coefficients for the two SPCs. Genes are sorted by their respective coefficients.

Figure S6

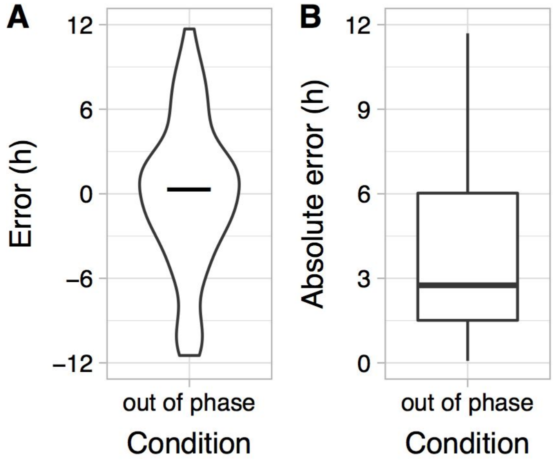


Ten-fold cross-validation (sumabsv=2, nSPC=2) on only the “out of phase” samples from GSE48113. Compare to Figure S5.

## Figure S7

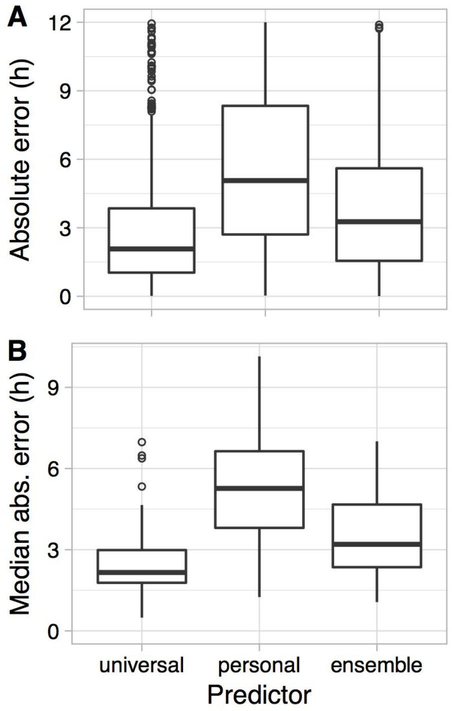


Boxplots of **(A)** absolute error and **(B)** median absolute error by individual for universal, personal, and ensemble predictors without universal guidance.

Figure S8

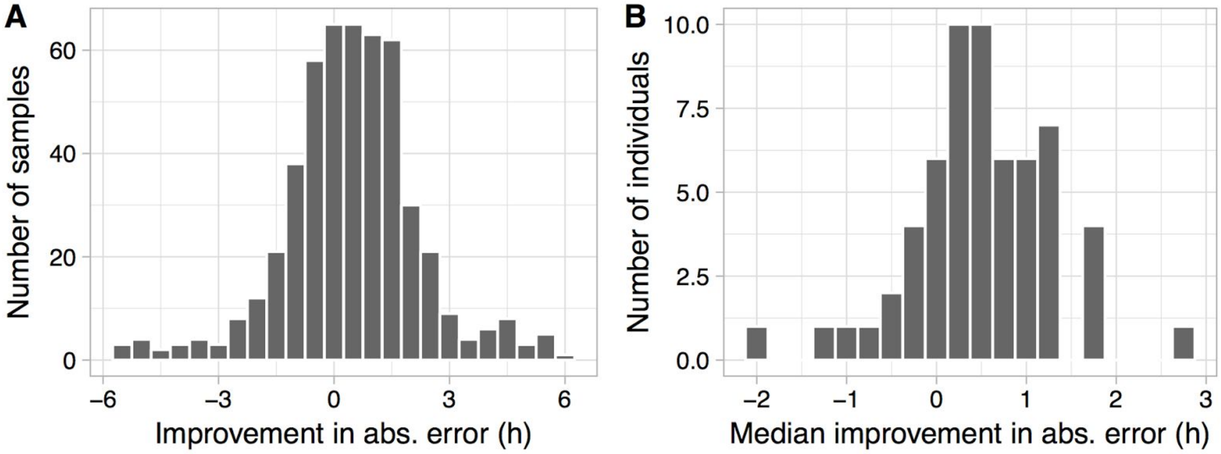


Histograms of **(A)** improvement in absolute error and **(B)** median improvement in absolute error by individual between the universal predictor and the ensemble predictor with universal guidance.

## Figure S9

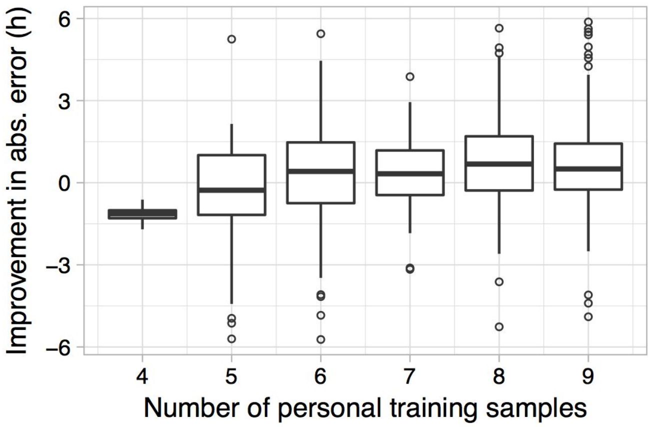


Boxplots of improvement in absolute error between the universal predictor and the ensemble predictor with universal guidance, as a function of number of personal training samples (because personal predictions were based on leave-one-out cross-validation, this is equal to the number of samples for the respective individual minus one).

## Figure S10

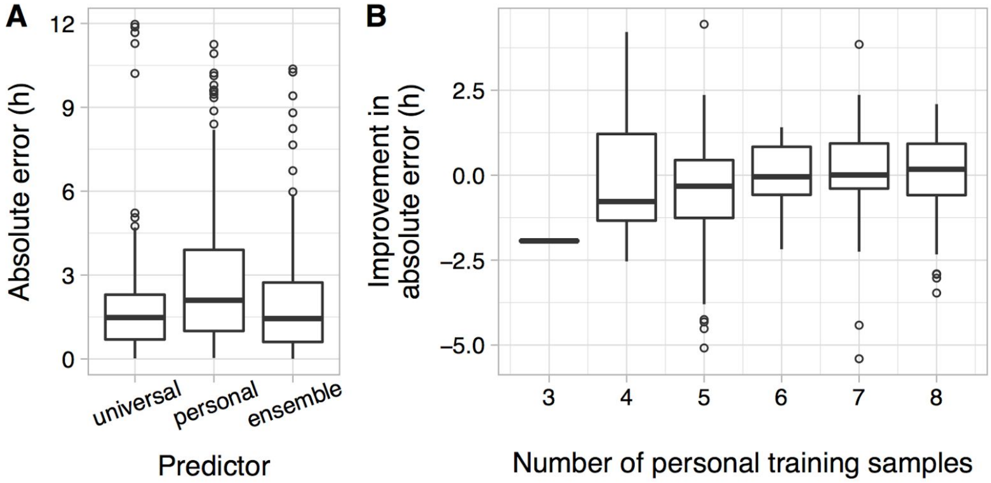


Personalized predictions with universal guidance applied to groups of samples. Each group consisted of two samples taken ~12 hours apart from the same individual. **(A)** Boxplots of absolute error for universal (standard 10-fold cross-validation, identical to Fig. 3), personal (leave-group-out cross-validation for each individual), and ensemble (circular mean of universal and personal) predictors. **(B)** Improvement in absolute error between universal predictor and ensemble predictor as a function of the number of personal training samples for that group (equal to the number of samples for that individual minus two).

Figure S11

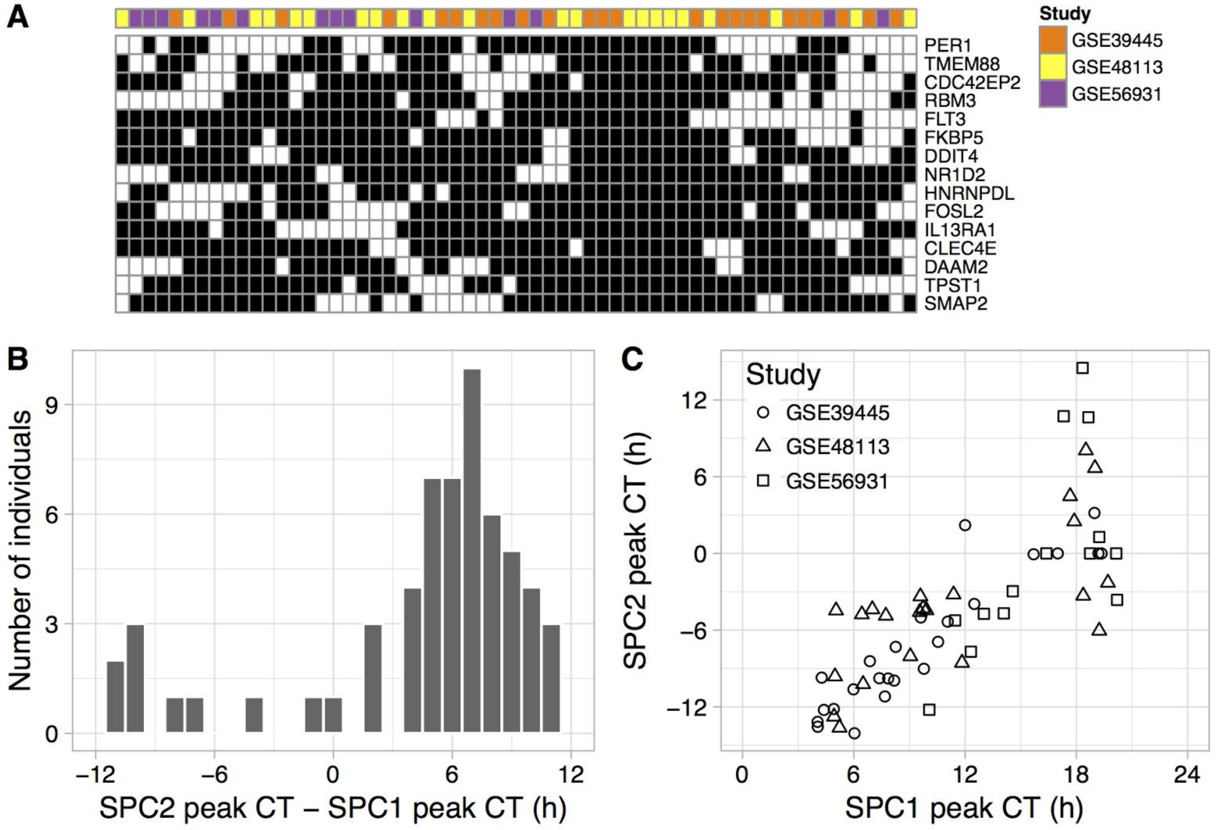


Genes and SPCs of the personal predictors trained with universal guidance. **(A)** Heatmap of genes present in personal predictors trained with universal guidance (using 15 genes present in predictor shown in Fig. 2). Rows correspond to genes and columns correspond to individuals. Black indicates the gene was present in the predictor for that individual. Rows and columns were sorted by hierarchical clustering. **(B)** Histogram of difference between peak times of SPC 1 and SPC 2. **(C)** Circadian times of peak expression for SPC 1 and SPC 2 in the personal predictors. Each point corresponds to one individual. For ease of visualization, some peak times for SPC 2 were shifted by 24 hours.
